# Influences of orientation on the Ponzo, contrast, and Craik-O’Brien-Cornsweet illusions

**DOI:** 10.3758/s13414-019-01953-8

**Published:** 2019-12-24

**Authors:** Leo Poom

**Affiliations:** Department of Psychology, Uppsala University, Box 1225, SE-751 42 Uppsala, Sweden

**Keywords:** Ponzo illusion, Simultaneous contrast, Craik-O’Brien-Cornsweet illusion, Orientation, Adjustment method

## Abstract

Explanations of the Ponzo size illusion, the simultaneous contrast illusion, and the Craik-O’Brien-Cornsweet brightness illusions involve either stimulus-driven processes (assimilation, enhanced contrast, and anchoring) or prior experiences. Real-world up-down asymmetries for typical direction of illumination and ground planes in our physical environment should influence these illusions if they are experience based, but not if they are stimulus driven. Results presented here demonstrate differences in illusion strengths between upright and inverted versions of all three illusions. A left-right asymmetry of the Cornsweet illusion was produced by manipulating the direction of illumination, providing further support for the involvement of an experience-based explanation. When the inducers were incompatible with the targets being located at the different distances, the Ponzo illusion persisted and so did the influence from orientation, providing evidence for involvement of processes other than size constancy. As defined here, upright for the brightness illusions is consistent with an interpretation of a shaded bulging surface and a 3D object resulting from a light-from-above assumption triggering compensation for varying illumination. Upright for the Ponzo illusion is consistent with the inducers in the form of converging lines being interpreted as railway tracks receding on the ground triggering size constancy effects. The implications of these results, and other results providing evidence against experience-based accounts of the illusions, are discussed.

## Introduction

**Fig. 1:**
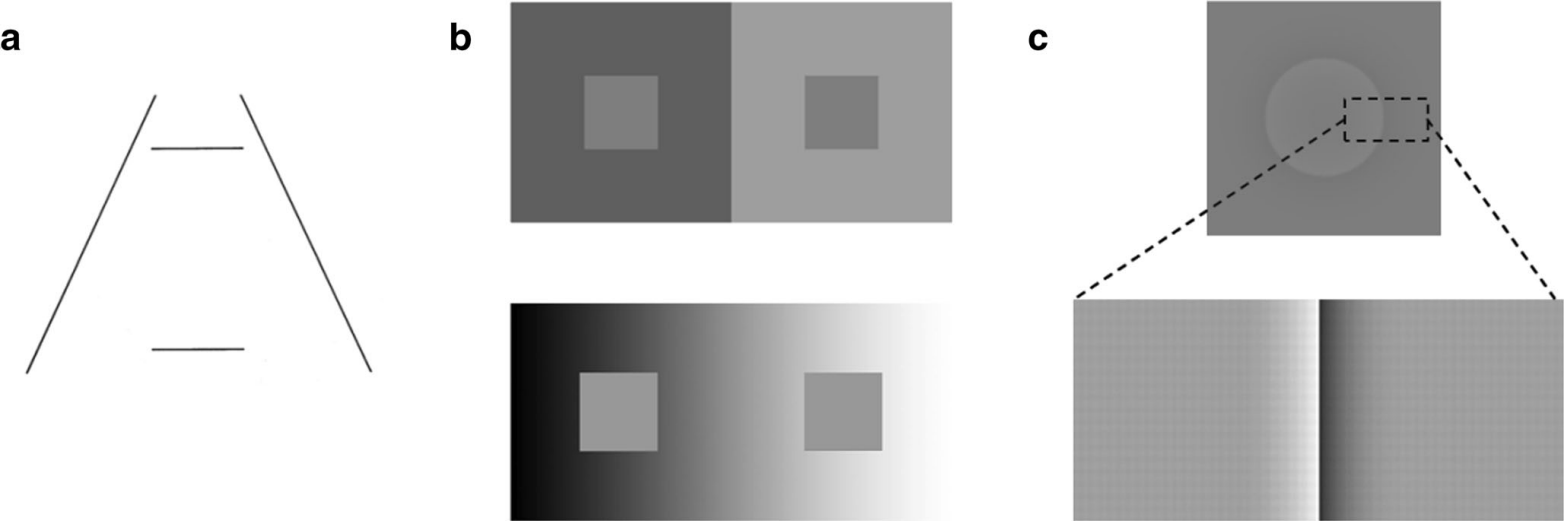
(**A**) The Ponzo illusion as originally demonstrated. The two horizontal lines have the same length but most observers perceive the upper one as longer. (**B**) Brightness contrast. **Top:** The two inset squares have the same luminance but the one embedded in a dark background is by most observers perceived as slightly brighter. **Bottom:** The luminance gradient providing a hint of a penumbra enhances the contrast illusion. (**C**) The Cornsweet illusion. **Top:** The original form. **Bottom:** As typically demonstrated with a horizontally oriented rectangular display, similar to the cut-out region of the original display with enhanced contrast. The left surface seems brighter than the right surface although they are same, which can be confirmed by covering the middle contrast with a pencil

Explanations of visual illusions can be categorized as either stimulus driven or experience based, and both types of explanations have received empirical support. Stimulus-driven explanations are contrast-enhancement mechanisms such as lateral inhibition, assimilation, and anchoring, and are typically orientation-invariant without adding additional assumptions. A central problem for the visual system, not explicitly dealt with by the stimulus-driven mechanisms, is the inverse optics problem (Berkeley, 1709/1976). von Helmholtz (1866/1924) suggested that experiences shape the input from the eyes, a process described as unconscious inference. Such empirically driven explanations predict influences of orientation on perceived illusion magnitudes. Studies investigating influences of orientation on perceived illusions are few. When it comes to size illusions, Rock (1984) made informal observations and claimed that the classical Ponzo size-illusion (Fig. 1A; Ponzo, 1911) remained unchanged when inverting the display. When it comes to lightness illusions I have found no published investigations of the influence of the orientation on the simultaneous contrast (Fig. 1B; which dates back to Alhazen, see history in Wade, 1996). Purves et al. (1999), however, investigated influences of orientation on the Craik O´Brien-Cornsweet lightness illusion (Fig. 1C; Cornsweet, 1970; Craik, 1940; O’Brien, 1958) and found that vertically oriented displays where the luminance contrast was consistent with shading caused by an assumption of illumination from above resulted in a stronger illusion than in the inverted condition. Here I investigate influences of orientation on illusion strengths for these three classical illusions: the Ponzo illusion, the simultaneous contrast illusion, and the Craik O´Brien-Cornsweet lightness illusion, henceforth labelled Cornsweet illusion for simplicity.

The Ponzo illusion is a geometrical-optical illusion where two identical target bars are seen as having different size due to inducing converging lines (Fig. 1A). The experience-based size constancy scaling theory is a frequently proposed explanation of the Ponzo illusion and many other geometrical illusions where in two-dimensional pictures cues that normally represent depth lead to distortion of perceptual experience (Gregory, 1963; Tausch, 1954). In the Ponzo illusion the converging lines, as typically results from everyday viewing of railway tracks or roads receding in depth, trigger size-constancy scaling (Gregory, 1963). So, images of objects of the same retinal sizes will look different if they appear to be at different distances, but Gregory noted that perceived depth was not required and that the size illusions occur even when the figure is seen to be flat. Gregory therefore assumed that constancy scaling operates whenever visual features associated with distance are detected, and can modify constancy scaling even when no depth is seen. So, the scaling can be set directly by depth features of flat figures, without any depth seen in the image (Gregory, 1963). This was also pointed out by Rock (1984), who noted that we do not necessarily perceive the inducing lines of the Ponzo illusion as receding in depth *“invert the figure or tilt it by 90° (by rotating the book): the impression of depth diminishes or disappears, but the magnitude of the illusion remains unchanged”* (Rock, 1984, p 156). Although the dominant explanation in textbooks for many geometrical illusions is based on size-constancy scaling, a number of variants of geometrical illusions have been presented that cannot be explained by such theories, and the size-constancy theory received early criticism (Hotopf, 1966). A number of variants of the Ponzo display have been presented that do not give any illusion or even reverse the illusion (Humphrey & Morgan 1965; Pressey, 1974a, b; Prinzmetal, Shimamura, & Mikolinski, 2001; Waite & Masaro, 1970). For example, if the horizontal lines in the classical Ponzo illusion are simply replaced by vertical lines, the illusion disappears, which is difficult to account for by the scaling theory (Humphrey & Morgan 1965; Waite & Masaro, 1970), and when successively increasing the angle between the inducing lines, the Ponzo illusion increases first but then disappears and finally reverses (Pressey, 1974a).

Alternatives to the experience-based size constancy theory are stimulus-driven explanations of geometrical illusions. The framing effect involves assimilation and enhanced contrast where objects that appear to fill an enclosing border appear larger than the same object surrounded by a larger frame (Girgus & Coren, 1982; Jordan & Randall, 1987). The upper bar in Fig. 1A closer to the bordering lines is therefore perceived as larger (assimilation) than the lower bar that is more distant from the oblique lines (contrast). An additional stimulus-driven theory claims that the Ponzo illusion, and other geometrical illusions, is caused by the misperception of orientation, similar to tilt illusions (Prinzmetal et al., 2001). It is assumed that the endpoints of the vertical lines constitute imagery lines whose orientations are repelled away from the inducing lines, similar to the classical tilt-contrast effect. Interestingly, this theory can account for the reversal of the Ponzo illusion occurring when increasing the apex angle between the inducing lines (Pressey, 1974a). Tilt repulsion and tilt attraction are actually the hallmarks of the classical tilt effect (Gibson & Radner, 1937) and seem to be mediated by different mechanisms (Wenderoth & Johnstone, 1988), which also generalize to other information-bearing media than luminance contrast to specify the tilted figures (Poom, 2000).

Among the most well-known brightness illusions are the classical simultaneous contrast illusion, first documented by Alhazen (circa 965–1040), and later by Helmholtz (1821–1894) and Hering (1834–1918) (as described in Wade, 1996), and the Craik-O’Brien-Cornsweet illusion (Cornsweet, 1970; Craik, 1940; O’Brien, 1958). The contrast illusion consists of two targets of equal luminance placed in inducing bright and dark backgrounds, respectively. The target located in the darker background is perceived as brighter than the one surrounded by the brighter background (Fig. 1B). The original form of the Cornsweet illusion (Fig. 1C, top) consists of an opposing luminance gradient meeting at an edge (an inducing discontinuous biphasic luminance edge) and forming a circular disc that produces a percept of a uniform brighter disc surface than the surrounding surface even though the luminance is actually the same. The Cornsweet illusion is usually displayed as a rectangular area with uniform gray separated by a vertical discontinuous biphasic luminance edge that creates the impression that one side of the flat luminance region is darker than the other region (Fig. 1C, bottom with enhanced contrast of the biphasic edge).

**Fig. 2:**
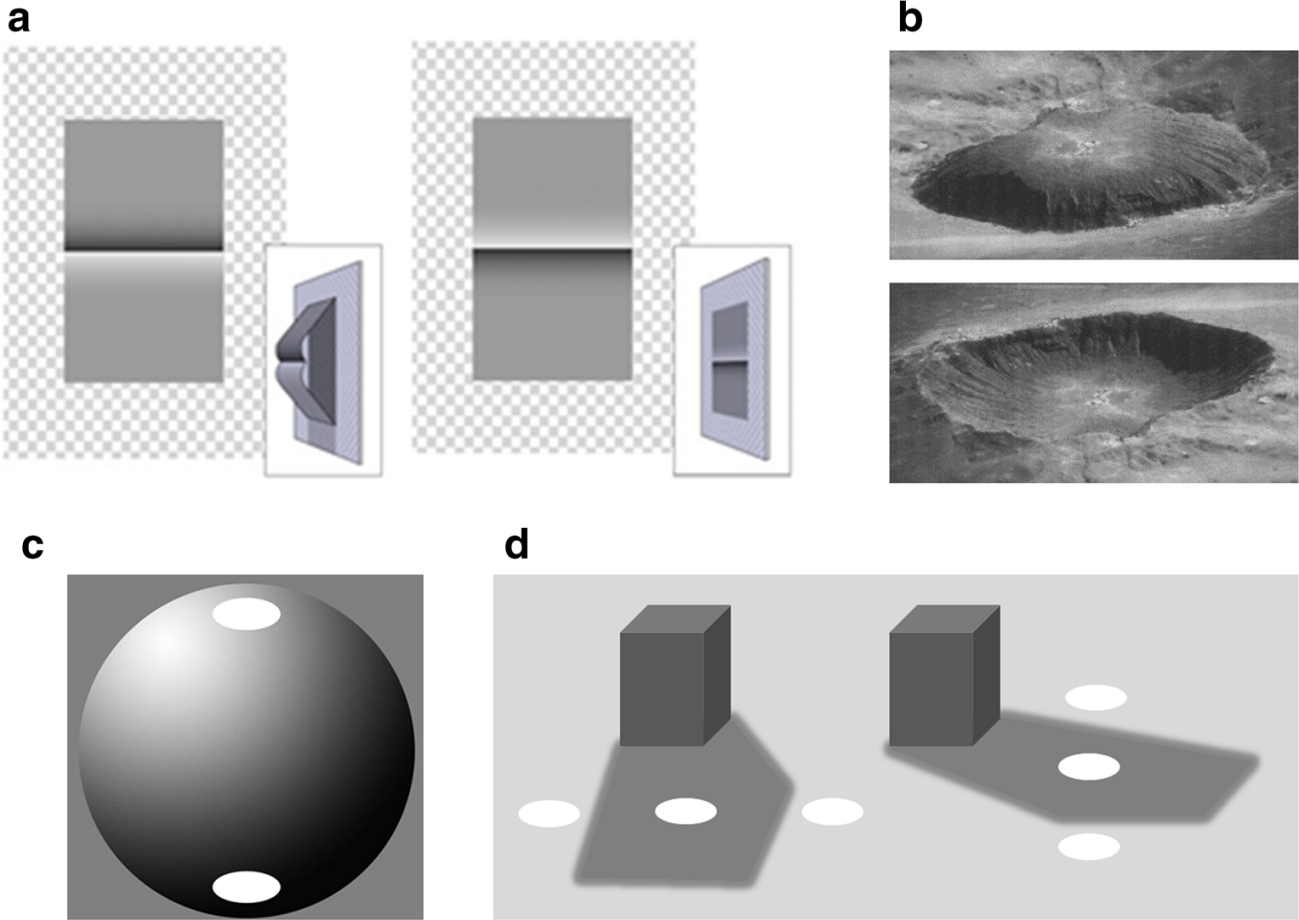
The illustrations show various shading scenarios given the light-from-above assumption. (**A**) The leftmost image is consistent with the 3D structure shown in the inset where the upper part is directly illuminated and the lower part is shaded. The rightmost image is an upside-down version of the left image and is consistent with the middle section being painted rather than shaded due to bulging (or as a sharp edge bulging outwards). The gray scales of the upper and lower parts of the surface now look more similar (from Purves et al., 1999. Copyright 1999, Society for Neuroscience). (**B**) The crater illusion; top-most image is an upside-down version of the bottom image. (**C**) Direct illumination occurs more frequently on top parts of objects than bottom parts, which more frequently receive indirect illumination. (**D**) When the shadow is due to cast shadows it is not so obvious how the shadowed and directly illuminated areas are likely to be oriented relative to each other. Still, combining these two causes of luminance variations would result in a greater frequency of occurrences of brighter regions on top. All discs in (**C**) and (**D**) have the same luminance, but the ones in the shadowed regions usually appear brighter

Empirical accounts of the Cornsweet illusion suggest the illusion arises due to prior encounters of sources of luminance and luminance gradients from shading in our 3D environment. In this interpretation, the direction of the luminance gradients is consistent with a bulging surface rather than a painted surface, as demonstrated in the leftmost part of Fig. 2A (from Purves et al., 1999). Although the two areas on either side of the luminance gradient are identical, the lower area appears brighter since it is consistent with being shaded if illuminated from above, triggering compensation of illumination by discounting the illuminant. The rightmost part of Fig. 2A illustrates the case when the image is inverted, and the light-from-above assumption is consistent with a flat painted surface where both areas are equally illuminated requiring no compensation of varying illumination. The light-from-above assumption constrains interpretations of shaded 3D objects and surfaces and is usually demonstrated by the crater illusion shown in Fig. 2B, where two possible depth interpretations in the crater illusion are resolved by using the light-from-above assumptions (Sun & Perona, 1998). In their study using the crater illusion as a probe, they also found a light-from-left bias, which seems to be influenced by cultural differences in reading/writing direction (Andrews et al., 2013).

The simultaneous contrast illusion may also be explained by compensation of spatially varying illumination levels. Since objects are more likely to be directly illuminated on surfaces facing upwards toward possible light sources, the luminance reflected from the top of objects is more likely to be more intense than in other parts that are more likely shaded. Another source of variations in illumination is cast shadows, in this case shadowed and illuminated ground regions could have any orientation relative to the observer depending on the relative positions of the shading object, the observer, and the light source. Combining these causes with luminance variation in images, we should expect influences of orientation on the simultaneous contrast illusion. Figure 2C and D illustrate this with shaded areas on 3D objects and cast shadows, respectively. This suggests that the contrast, the Cornsweet, and the crater illusion all arise from separating illumination from surface reflectance. Accordingly, adding information about illumination and shading enhances the simultaneous contrast and Cornsweet illusions (Purves et al., 1999; Williams et al., 1998). This is illustrated in Fig. 1B, bottom image, where the discrete contrast of the background luminance is replaced by a continuous luminance gradient, similar to a penumbra, which enhances the appearance of shading (Bergström, 1977) and increases the contrast illusion (Fig. 1B).

Evidence against the discounting illumination explanation comes from Todorović (2006), who showed that images that were shadow-incompatible but otherwise similar to the contrast and Cornsweet displays still produced strong brightness illusions. These demonstrations provide strong evidence against an experience-based explanation where the visual system discounts the illumination level. Todorović (2006) provided evidence that processes relying on local luminance contrasts and gradients surrounding target regions could better explain lightness illusions. Among such stimulus-driven explanations of brightness illusions is spatial filtering by lateral inhibition. This process discounts the illumination and enhances perceived local contrast (Anstis et al., 1978; Cornsweet, 1970). Such filtering combined with assimilation of lightness across enclosed areas, or amplification of low spatial frequency structure of the image (Dakin & Bex, 2003; Hong & Grossberg, 2004), explains both the contrast effect and the Cornsweet illusion (e.g., Davey et al., 1998; Grossberg & Todorović, 1988; Komatsu, 2006; Todorović, 1987, 2006). In addition, lateral inhibition has been proposed as a common mechanism for various contrast-amplifying effects such as from orientation/tilt (Blakemore, Carpenter, & Georgeson, 1970; Gibson, 1937; Poom, 2000; Poom et al., 2007), motion (Anstis & Casco, 2006; Duncker, 1929/1938), spatial frequency/size (Klein, Stromeyer, & Ganz, 1974), depth (Graham & Rogers, 1982), luminance contrast (Chubb, Sperling, & Solomon, 1989), chromatic saturation/contrast (Brown & MacLeod, 1997), blur (Webster, Georgeson, & Webster, 2002), the Cornsweet illusions for texture density (Mackay, 1973), line length (Crovitz, 1976), visual depth (Anstis et al., 1978), and contrast (Lu & Sperling, 1996). More complex filtering has been proposed to account for a number of other brightness perception phenomena (Blakeslee & McCourt, 2004).

Other stimulus-driven explanations for the contrast effect are based on the finding that perceived brightness involves estimating relations between luminance values across the scene (Wallach, 1948) where the highest luminance values are assigned a standard anchoring lightness of perceived white (a form of lightness balancing). This idea has been extended by assuming that separate standards are used in separate fields of illumination (Katz, 1935), or in separate areas formed by Gestalt principles. Separate anchoring processes are then applied to these regions (Gilchrist, 2014). Within each region the visual system assumes that the highest luminance value is white and uses it as a standard for the interpretation of other luminance values in that region (Li & Gilchrist, 1999). Cross-talk between multiple local regions and the global region as proposed by Kardos (1934) has the potential to explain the contrast illusion and multiple other luminance-based illusions (such as the Benary effect, the Whites illusion, the Todorović illusion, the Bressan illusion, and the reversed contrast illusion; Gilchrist, 2014). In the stimulus-driven vector-model (Bergström, 1977), perceived illumination is separated from perceived reflectance by the assumption (possibly arrived from experience) that local stepwise luminance gradients inform about contrasts from painted surfaces whereas gradual gradients inform about illumination and three-dimensional form (reflectance is influenced by surface orientation relative to the observer). This relatively simple vector-model can account for a variety of different lightness phenomena including simultaneous contrast and the classical Cornsweet illusion.

The stimulus-driven explanations (lateral inhibition, assimilation, anchoring, or framing effects), as originally formulated, do not predict that the orientation of the illusion display should influence the illusion in any way. Elaborated stimulus-driven mechanisms, however, may be influenced by experience such that, for example, neural connections are strongest for particular orientations. If, on the other hand, illusions arise as a consequence of our past experiences, then influences of orientation are certainly expected since our environment is typically asymmetrical in the vertical direction (i.e., sky vs. ground, locations of typical light sources are typically above us, and how gravitation influences our environment). Illusions that build upon such asymmetrical past experiences should then depend on the orientation of the image. If the Ponzo, the simultaneous contrast, and the Cornsweet illusions rely on common processes such as lateral-inhibition and assimilation alone, we should expect correlations between illusion magnitudes across illusions. Correlations between illusions are also expected if influences from experiences alone are the dominant cause and if the weight put on these experiences is an individual specific characteristic that generalizes across stimulus dimensions. Importantly, the light-from-above assumption provides clear predictions of influences of orientation on the Cornsweet illusion and on the contrast illusion, and therefore may provide evidence for the involvement experience in the production of these illusions. Similarly, physical asymmetries between spatial directions up and down should manifest themselves in influencing the magnitude of the Ponzo illusion, depending on its orientation.

## Experiment 1

### Methods

A task that measures the strength of visual illusions across many conditions should be reliable with a limited number of trials per condition. The method of adjustment (MA) was used to fulfill this requirement. When using the MA observers adjust the level (here brightness or size) of an adjustable part of the stimulus to obtain the point of subjective equality (PSE) to a corresponding fixed part of the stimulus (test stimulus). This is repeated multiple times and the strength of an illusion is then estimated from the average difference between the PSE and the point of objective equality.

### Participants

Thirty participants (19 females) aged between 19 and 65 years (mean = 29 years, SD = 12) were recruited, all right-handed except one, and they were either granted experimental credit or given a cinema ticket for compensation (value approx. 10 euro). They all had normal or corrected-to-normal vision. The sample size was based in part on available resources and from considering previous experiments in this field where typically smaller samples have been used and effect sizes expected are relatively large.

### Stimuli

**Fig. 3:**
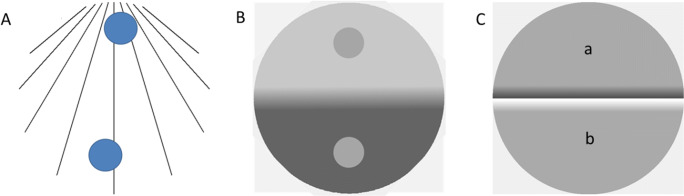
Stimuli used in the experiments, all shown with 0° orientation. (**A**) The Ponzo-illusion: the circular discs are the same size but people tend to perceive the upper one as larger. (**B**) Brightness contrast: The two small discs are equally bright but most people perceive the upper one as darker. (**C**) The Cornsweet illusion: The surface labeled **a** seems to be darker than the surface labeled **b**, although they are same

Figure 3A, B, and C shows examples of the Ponzo illusion, brightness contrast illusion, and the Cornsweet illusion as used in the present study. The PSE was measured for eight different orientations of the stimulus displays: 0°, 45°, 90°, 135°, 180°, 225°, 270°, and 315°. Cardinal orientations are the two displays with symmetry around the vertical axis (0° and 180°) and the horizontal axis (90 and 270°). Henceforth the 0° orientation is referred to as the upright display and the 180° orientation the upside-down illusory display. Figure 3A, B, and C shows a 0° upright version of the Ponzo, simultaneous contrast, and Cornsweet display as used in this experiment. The contrast illusion is created with a penumbra separating the bright and dark background to increase the illusory strength (Williams et al., 1998). Measures of the magnitudes of the illusions were obtained by adjusting the homogenous brightness regions of part a or b in the Cornsweet stimulus, one of the circles in the simultaneous contrast stimulus, or the size of one of the circles in the Ponzo stimulus to obtain a PSE compared to the opposite area or test circle. All stimuli were shown within a circular area of 12.3° as viewed from 60 cm distance from the screen. The background luminance of the Cornsweet and the simultaneous contrast stimuli was gray with 5 cd/m^2^, and the background luminance of the Ponzo stimuli was 65 cd/m^2^.

The Ponzo illusion was induced by nine black converging lines within the circular stimulus area to give an impression of linear perspective. Line separation was doubled for each visual degree along the density gradient. On each side along the lines was a disc with its center located 1.8° within the border of the stimulus area. To avoid alignment, the discs were laterally displaced relative to the symmetry axis; their lateral displacement was drawn from a uniform distribution of ±1.5° (see Fig. 3A). One of the discs had a fixed diameter of 2.5°. The initial setting of the other adjustable disc diameter was randomly assigned from a uniform distribution: 2.5° ± .35°. The luminance of the discs was 110 cd/m^2^ and the color was yellowish (RGB code was (255, 191, 128)).

The simultaneous contrast stimuli were created by dividing the stimulus area into two semicircles with luminance 30 cd/m^2^ and 100 cd/m^2^, respectively. A smooth luminance gradient with a width of 2° separated the two semicircles (informal inspection revealed that the illusion appeared much stronger with a gradient than with a discontinuous sharp border between the semicircles). On each semicircle was a disc with a 2° diameter, one with fixed luminance of 65 cd/m^2^ and the other with an adjustable luminance level. Initial luminance of the adjustable disc was randomly assigned from a uniform distribution 30–100 cd/m^2^.

**Fig. 4:**
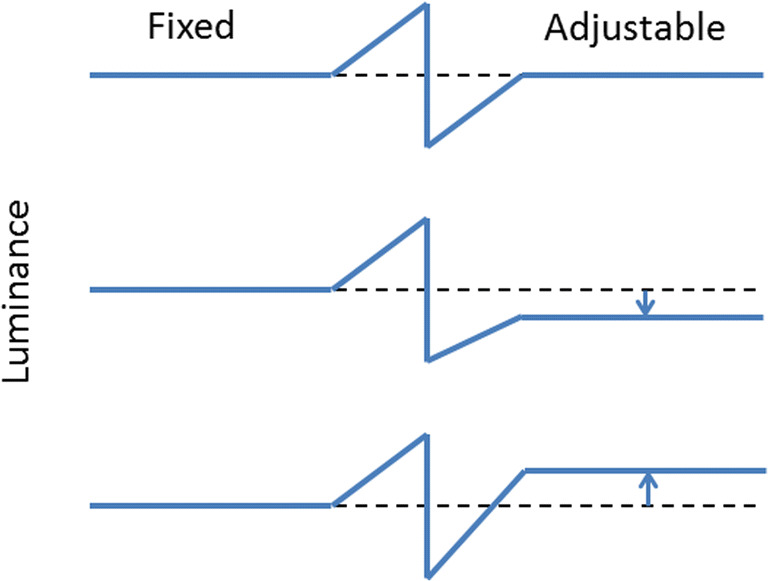
Cross-section schematically showing the luminance profile of the Cornsweet illusion (**top**), and how the setting of luminance level of the adjustable side to obtain subjective equality with the fixed side influenced the slope of the luminance gradient on the same side

The Cornsweet stimulus consisted of two semicircles, one with fixed luminance and the other with adjustable luminance. The semicircles met at a semi-phasic luminance edge (a positive and a negative luminance gradient meeting at a discontinuous luminance edge); each such gradient had a width of .8°. Across the discontinuous gap, the luminance ranged from 5 to 170 cd/m^2^. The semicircle with a fixed gray was 85 cd/m^2^. Initially, the luminance of the adjustable semicircle was randomly assigned from a uniform distribution between 40 and 130 cd/m^2^. When adjusting the luminance level, the luminance gradient was co-adjusted to merge with the luminance level of the uniform adjustable area as schematically illustrated in Fig. 4.

### Design

**Fig. 5:**
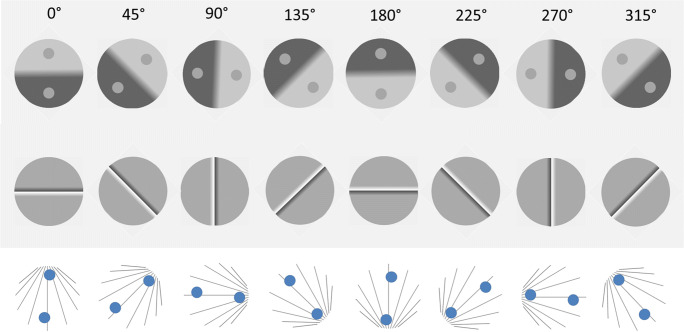
Displayed are all stimulus orientations used for the three illusions. From top to bottom row: Simultaneous contrast, Craik-O´Brien-Cornsweet, and the Ponzo illusion

Each illusion was presented in eight orientations (Fig. 5), each repeated eight times and randomly interleaved. The three illusions were blocked but the order was randomized between participants. For half the trials the initially brighter side of the Cornsweet illusion, the disc over the brightest background in the brightness contrast illusion, and the disc over the denser part of the converging background lines in the Ponzo illusion were adjustable. For the rest of the trials the other side/disc was adjustable (these two conditions were collapsed). This resulted in an 8 × 8 trials repeated-measures design for each illusion.

### Analysis

Illusion magnitudes estimated as proportions of deviations of subjective equal from physically equal were calculated as the positive or negative difference between the fixed and the adjusted stimulus divided by the fixed stimulus [magnitude = ± (fix-adj) / fix]. When, according to the prediction of the illusion, the fixed side is larger/brighter, then the positive sign is used, and when the fixed side is smaller/darker, then the negative sign is used. In short, this measure resulted in magnitudes that were larger than zero when illusion was in the expected direction. Percentage of illusion strength is obtained by taking the proportion magnitude times 100.

Statistical tests were performed by the freely available statistical software JASP (JASP Team, 2019), which allows calculation of both p-values and Bayes factors, which are both presented. The Bayes factor BF_10_ is the ratio between the probabilities of the results given H_1_ and H_0_ and is thus a measure of the relative evidence in favor of H_1_. Bayes factors are more intuitive than p-values and have several other advantages (Wagenmakers et al., 2018). For example, unlike the p-value, the Bayes factor can be interpreted as strong evidence in favor, or against, the null hypothesis (H_0_). All reported Bayes factors were computed using the default settings in JASP for the effect size priors (Cauchy scale parameter = 0.707; r scale for fixed effects = 0.5).

## Results

### Influence of orientation on illusion magnitude

**Fig. 6:**
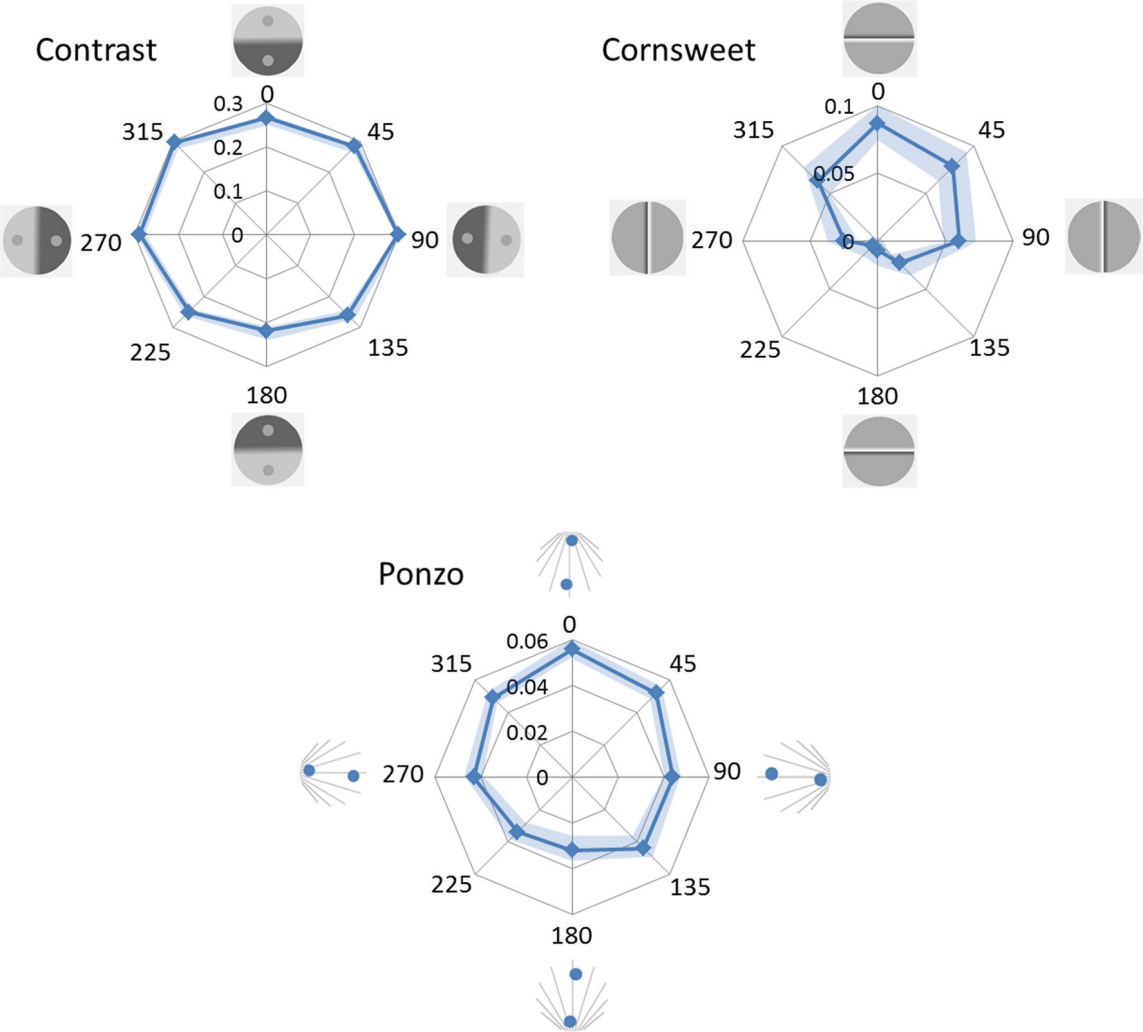
The radar plots show the mean illusion magnitude expressed in proportions as a function of orientation in degrees from 0° to 315°. A positive sign indicates that the illusion is in the expected direction. Presented along the cardinal orientations (0°–180° and 90°–270°) are illustrations of stimuli. The shaded areas represent the 95% confidence intervals

Radar plots in Fig. 6 show the mean of the illusion magnitudes calculated from the PSEs as a function of orientation for each illusion.

Table 1 shows the results from an analysis of influence of orientation from separate ANOVAs for each illusion. Both p-values and BF_10_ are presented. For the contrast and the Cornsweet illusions, the results are about 10^16^-10^17^ times more likely given an influence of orientation than if there was no such influence. For the Ponzo illusion the corresponding BF_10_ is 492. The results present clear evidence for an influence of orientation for all three illusions.

**Table 1:** Influences of orientation on illusion magnitudes

Illusion	F (7,203)	P	BF_10_	η^2^
Contrast	15.3	<.001**	1.2∙10^16^**	.345
Cornsweet	20	<.001**	1.8∙10^17^**	.409
Ponzo	4.9	<.001**	492**	.145

**p < .001, BF_10_ > 100

Table 2 shows comparisons of illusion strengths between opposite pairs of orientations: 0°–180°, 45°–225°, 90°–270°, 135°–315°, respectively. The contrast illusion was influenced by orientation, the PSE indicated 27% compensation in the upright condition and 22% compensation in the upside-down condition. The illusion magnitude for the opposite horizontal orientations was almost identical (30% in 90° orientation and 29% in the 270° orientation condition).

**Table 2:** Post hoc comparisons. Mean differences are positive when the illusion is stronger: for upright (0°) compared to upside-down versions (180°), when tilted 45° clockwise compared to 225°, when tilted 90° clockwise compared to 270°, and when tilted 315° clockwise compared to 135°

	Orientation pair	Mean difference (SE)	t(29)	p_bonf_^†^	BF_10, U_^†^
Contrast	0–180°	.047 (.013)	3.53	.040*	24*
	45–225°	.036 (.010)	3.50	.043*	23*
	90–270°	.010 (.008)	1.19	1.00	.37
	315–135°	.035 (.008)	4.24	.006*	135**
Cornsweet	0–180°	.081 (.012)	6.59	<.001**	50,580**
	45–225°	.074 (.013)	5.76	<.001**	6,188**
	90–270°	.036 (.010)	3.70	.025*	36*
	315–135°	.040 (.012)	3.33	.066	16*
Ponzo	0–180°	.024 (.006)	3.76	.022*	41*
	45–225°	.018 (.006)	2.83	.235	5.2
	90–270°	.00073 (.005)	.141	1.00	.20
	315–135°	.006 (.007)	.79	1.00	.26

*p < .05, BF_10_> 10

**p<.001, BF_10_> 100

^†^Bonferroni-corrected p-values based on all 24 pairwise combinations of the eight orientations are presented; the subscript U denotes that the BFs are un-corrected

The Cornsweet illusion was strongly influenced by orientation: the illusion magnitude was 8.7% in the upright condition compared to .09% in the upside-down condition. The Cornsweet illusion also differed in magnitude between the opposite orientations along the horizontal. When the display was oriented 90°, illusion magnitude was 6%, compared to 2.5% illusion magnitude in the 270° condition. This is contrary to demonstrations of a light-from-left bias in disambiguating shape from shading (Sun & Perona, 1998). In the lab a weak light source on the participants’ right side was used, which might have biased the results along the vertical as found here; this hypothesis is further examined in Experiment 2.

The Ponzo illusion was also influenced by its orientation: the PSE indicated 5.6% illusion magnitude in the upright condition compared to 3.2% in the upside-down condition. The illusion magnitudes for the opposite horizontal orientations were almost identical (4.4% in the 90° condition and 4.3% in the 270° condition).

### Correlations of illusion magnitudes between orientations

Strong correlations between individual illusion magnitudes across orientations were obtained within illusion demonstrating a high reliability of the PSE measures. Correlations between PSE settings were found for all pairs of orientations for the Cornsweet (average: r = .64, range: .45 < r < .88, all ps < .006, 4.6 < BF_10_ <5∙10^7^) and contrast illusions (average: r = .81, range: .63 < r < .93, ps < .001, 160 < BF_10_ < 2.8∙10^10^). The PSE settings for the Ponzo illusion were also correlated but varied more across orientations between individuals (average: r = .37, range: -.049 < r < .84, .001 < p < .60, .23 < BF_10_ < 1.33∙10^6^).

**Fig. 7:**
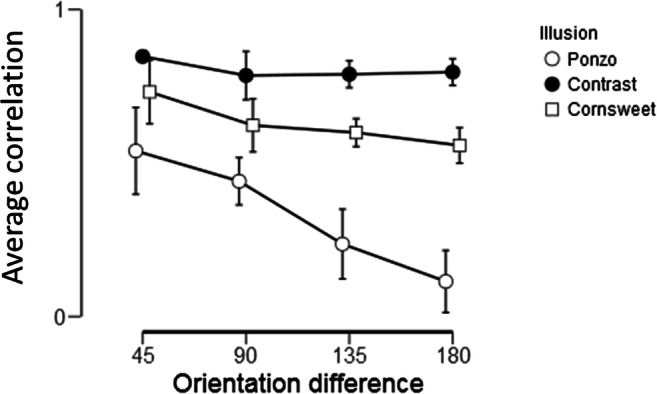
The average correlations between PSE (illusion strength) as a function of the absolute difference between orientations for each illusion. The 95% CIs are displayed

Next within-illusion correlations as a function of orientation difference were analyzed. For each orientation O_i_, the correlations of illusion magnitudes obtained between O_i_ and +/-45°, O_i_ and +/-90°, O_i_ and +/-135°, O_i_ + 180° (note that O_i_ +/- 180° is one and the same). Fisher’s z-transform [z = arctanh(r)] was applied to the correlations to obtain approximate normal distributed values for the repeated-measure ANOVA (illusion ∙ orientation difference). In the Bayesian analyses BF_inclusions_ were calculated, which compare models that contain the effect to equivalent models stripped of the effect, and where higher-order interactions are excluded. Figure 7 and Table 3 show that correlations between illusion strength decreased systematically as a function of orientation difference (simple main effects for the Cornsweet, BF_10_ = 83.6, and for the contrast illusion, BF_10_ = 19756), no reliable decrease from the contrast-illusion was obtained (BF_10_ = 1.35). Main effects of illusion were found (F(2, 14) = 74.2, p<.001, η^2^ = .91, BF_inclusion_ ≈ ∞). Largest correlations between orientations for all orientation differences were found for the contrast-illusion, followed by the Cornsweet-illusion and the Ponzo illusion. In addition, a main effect of orientation difference was found (F(3, 21) = 30.6, p < .001, η^2^ = .81, BF_inclusion_ = 2.7 ∙ 10^6^) and an interaction between illusion and orientation difference (F(6, 42) = 2.96, p = .017, η^2^ = .30, BF_inclusion_ = 9.5).

**Table 3:** The results of an ANOVA investigating influence of orientation difference on correlations (z-transformed) between illusion magnitudes, showing p-values, and Bayes factors from simple main-effect analyses for each illusion, N = 30

	F(3)	P	BF_10_
Cornsweet	7.8	<.001**	83.6*
Contrast	2.6	.080	1.35
Ponzo	18	<.001**	19,756**

*p < .05, BF_10_ > 10

**p <=.001, BF10 > 100

### Any common factor between illusions?

No common factor for susceptibility across illusions was found for either the absolute values of PSEs or influences of orientation on PSEs. Table 4 shows the correlations between the absolute values of PSEs (averaged across all eight orientations for each illusion) and correlations between the average influences of orientation on illusions for each pair of illusions. For the average magnitudes the BF supports H_0_ (BF_01_ = 1/ BF_10_); the results are seven to nine times more likely under H_0_ (there is no correlation) than under H_1_ (there is a correlation). Influence of orientation was calculated from the difference in PSEs obtained between upright and upside-down versions, i.e., 0° and 180°, and differences in opposite oblique orientations, 315°–135° and 45°–225°, of each illusion and then averaged. These opposite orientations resulted in the largest differences across all three illusions.

**Table 4:** Pearson correlations between average illusion magnitudes across illusions, and correlations of orientation influence across illusions averaged across differences between opposite vertical (0°–180°) and oblique orientations (315°–135°, 45°–225°)

	Average magnitudes*			Influence of orientation*		
	Pearson's *r*	p	BF_10_	Pearson's *r*	p	BF_10_
Cornsweet-Contrast	-.124	.74	.15	.238	.10	.49
Cornsweet-Ponzo	-.139	.77	.14	.071	.35	.24
Contrast-Ponzo	-.238	.90	.11	-.247	.91	.52

*One-tailed for positive correlation

## Experiment 2

Unexpectedly, in Experiment 1 a stronger Cornsweet illusion was obtained when the display was oriented 90° compared to 270°. This vertical asymmetry could be consistent with a hypothetical light-from-right assumption, which is contrary to the light-from-left assumption previously found (Sun & Perona, 1998). It has been shown that the direction of light assumption can be modified from experience (Adams, Graf, & Ernst, 2004), and it is possible that the direction of the weak illumination from the right side relative the participant, as used in the laboratory, might have influenced the participants’ perception of the Cornsweet illusion. Experiment 2 was performed to test the above-mentioned hypothesis about possible influences of left versus right light direction on the Cornsweet illusion using a glare effect presented on the computer screen. Also, Experiment 2 serves as a test to replicate the influence of orientation, without manipulating illumination as found in Experiment 1.

The glare effect is a brightness illusion in which a white central region appears self-luminous when surrounded by linearly decreasing luminance ramps (Zavagno & Caputo, 2001), and gives rise to the perception of self-luminosity, or glow with low light emission, in the case of computer-generated displays (Todorović, 2006). Trials with glare on either the left or right side of the computer screen were interleaved with trials presenting no glare. It was hypothesized that that the glare would influence the illusion magnitude for the horizontally oriented displays (90° and 270°) in opposite directions depending on which side the glare is shown. The illusion should be symmetric for the two opposite horizontally oriented displays when no glare is presented.

### Method

As in Experiment 1, the method of adjustment (MA) was used but only the two brightness illusions were investigated: the contrast and the Cornsweet illusions.

### Participants

Thirty participants (17 females) aged between 21 and 42 years (mean = 29 years, SD = 5) were recruited, all were right-handed except one. They were either granted experimental credit or given a cinema ticket for compensation (approx. value 10 euro). All had normal or corrected-to-normal vision.

### Stimuli

**Fig. 8:**
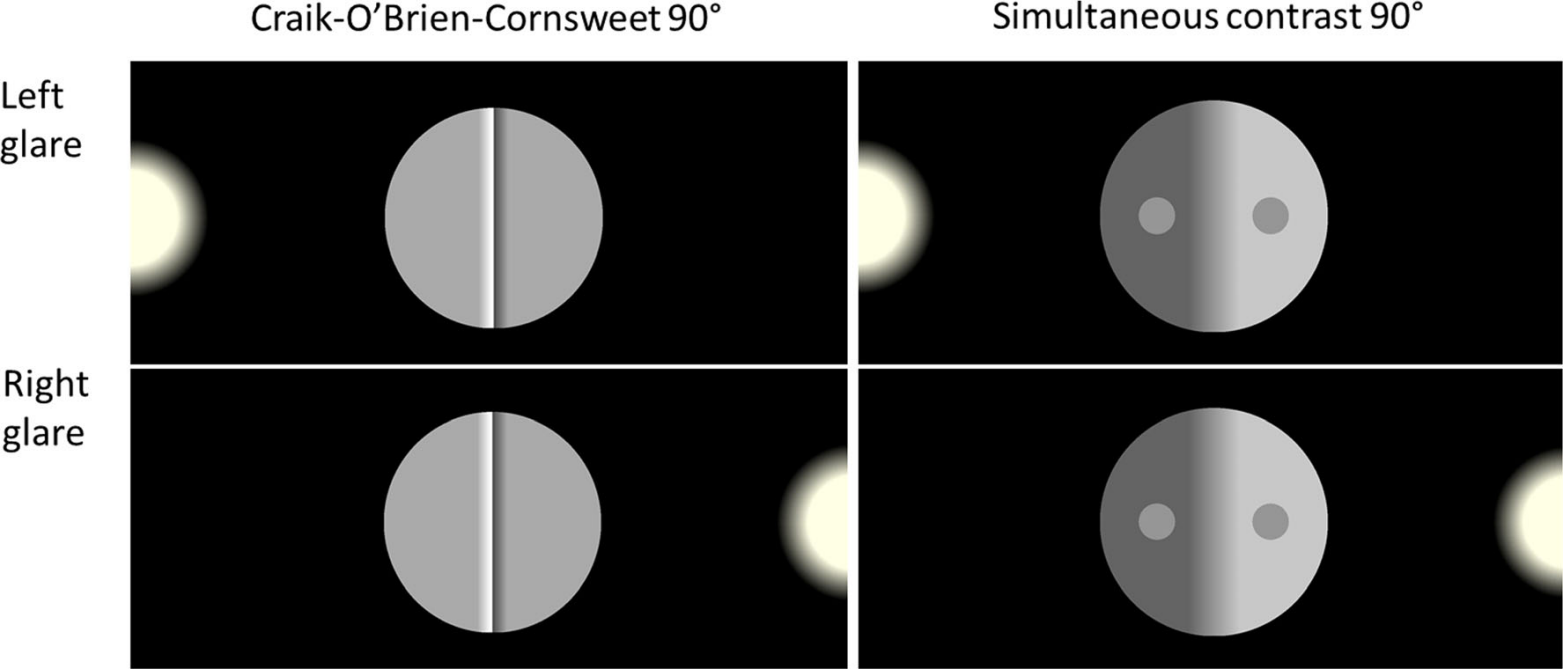
Displays used in Experiment 2 showing the left-glare and right-glare conditions for the two illusions, here oriented 90° in all four displays. The two right-glare panels at the bottom are consistent with an interpretation of shading resulting from 3D shapes. The two left-glare panels on top are less consistent with a similar interpretation

Stimuli were the same as in Experiment 1, but only four orientations were used (vertically oriented 0° and 180°, and horizontally oriented 45° and 370°). In a subset of randomly interleaved trials, a glare was presented on the right or the left side of the computer screen in the 90° and 270° orientation conditions (Fig. 8), placed straight behind the observer was a weak real light source.

### Results

To test the light-direction hypothesis separate repeated-measure (2 × 2) analyses were performed for the two illusions with two independent factors Light (left vs. right) and Orientation (90° vs. 270°).

**Fig. 9:**
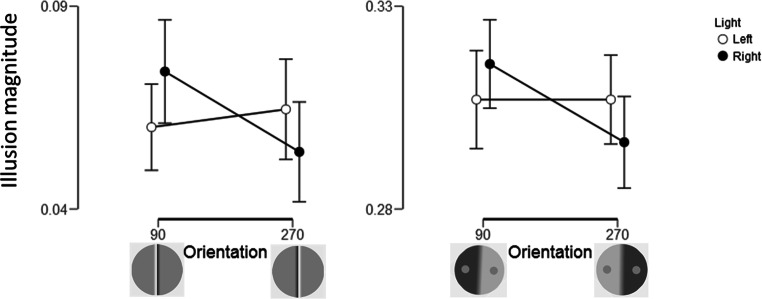
Illusion magnitude as a function of stimulus orientation and direction of incoming light from the simulated glare on the screen. The 95% CIs are displayed

Figure 9 shows the results. Interactions between Light and Orientation were obtained for both the Cornsweet (F(1, 29) = 12.7, p = .001, η^2^ = .30) and the contrast illusion (F(1, 29)= 5.53, p = .026, η^2^ = .16) as indicated by the p-values, although BF_inclusions_ 1.48 and .94, respectively, were not that impressive. No main effects of light or orientation were obtained for the Cornsweet illusion (Light: F(1,29) = .24, p =.63, BF_inclusion_ =.20; Orientation: F(1, 29) = .73, p = .4, BF_inclusion_ = .40), or the contrast illusion (Light: F(1, 29) = .025, p = .88, BF_inclusion_ = .16; Orientation: F(1,29) = 2.1, p = .16, BF_inclusion_ = .94). The BF_inclusions_ provided at most weak support for the absence of these effects.

To investigate in more detail the influence of light direction on the illusions, paired-sample two-tailed t-tests were performed (Table 5). In line with the hypothesis, the glare manipulations influenced the Cornsweet display most, specifically when oriented 90°. For both illusions, the target on the other side of the simulated light appears brighter. So, this influence of the glare-direction cannot be due to a misperceived decaying gradient of perceived lightness across the screen originating from the glare.

**Table 5:** The results from t-tests on the influence of left vs. right light direction on perceived illusion magnitudes, analysed separately for each illusion-orientation for the two illusions

	Light direction diff., orientation	t(29)	p	Effect size	BF_10_
Cornsweet	Left-Right, 90	-3.3	.003*	-.60	14.3*
	Left-Right, 270	1.9	.056	.36	1.10
Contrast	Left-Right, 90	-1.2	.23	-.22	.39
	Left-Right, 270	1.6	.13	.29	.59

*p < .05, BF_10_ > 10

**Fig. 10:**
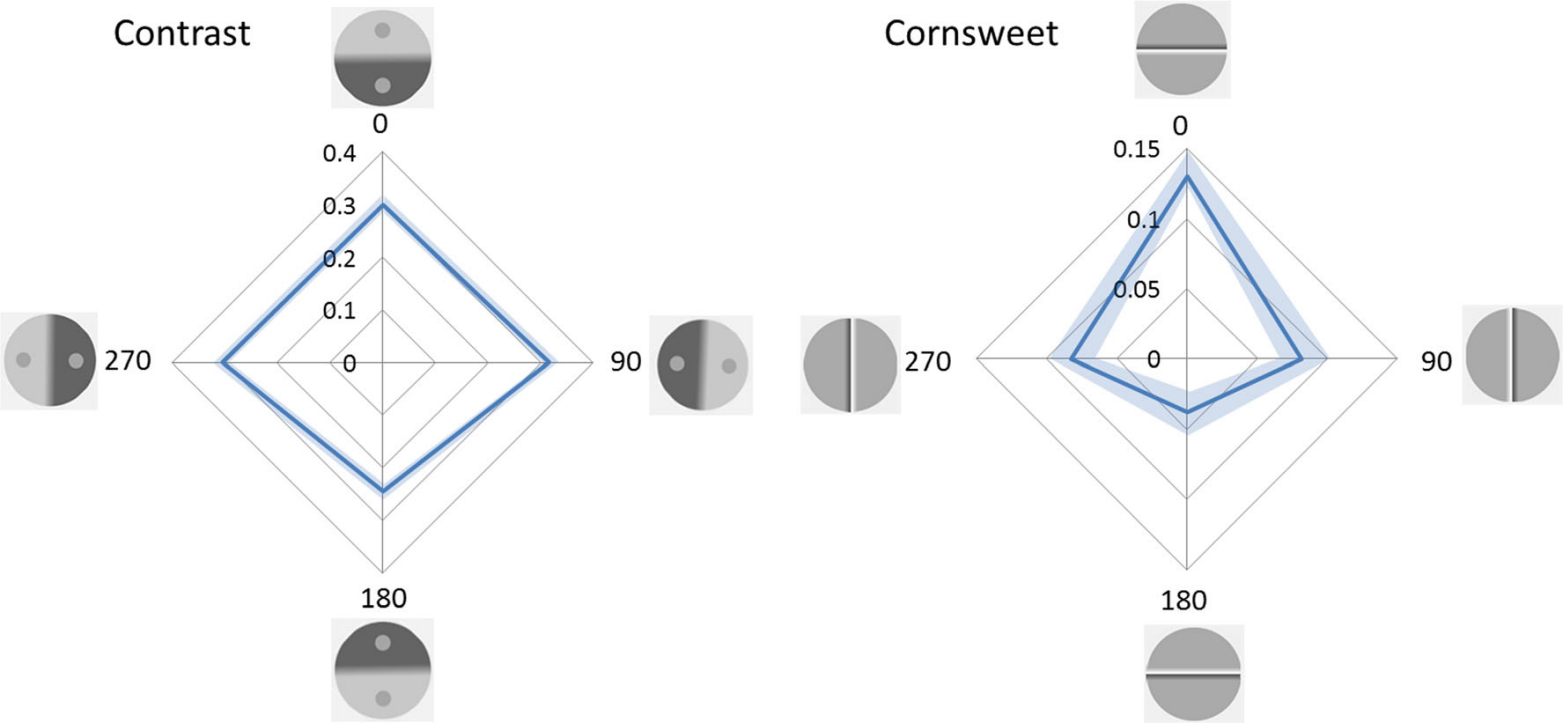
The radar plots show the mean illusion magnitudes for each of the orientations in the no-glare conditions. The shaded areas represent the 95% CIs

From Fig. 10 it is clear that no difference in PSE was found between the 90° and 270° orientation conditions in no-glare conditions for the Cornsweet (8.1% and 8.3% illusion magnitudes, t(29)= -.1, p= .92, d= -.018, BF_10_ = .19) or the contrast illusion (31.7% and 30.5% illusion magnitudes, t(29)= 1.9, p=.064, d=.35, BF_10_=.99). Thus, the increased illusion for the Cornsweet illusion oriented 90° compared to 270° as found in Experiment 1 was not obtained in Experiment 2. These results provide support for the hypothesis that the vertical asymmetry of the Cornsweet illusion found in Experiment 1 was caused by the low-level light in the lab that was located at observers’ right side, although the simulated glare might not have been ideal for this purpose.

As found in Experiment 1, both illusion magnitudes differed between displays oriented 0° and 180° (Fig. 10, Cornsweet: 13% and 3.8% illusion magnitudes, Contrast: 30.1% and 24.5% illusion magnitudes). The group-level difference of the contrast illusion showed that the 0° orientation resulted in a stronger illusion than the 180° display (t(29) = 4.8, p< .001, d =.89, BF_10_ = 690), and the 0° Cornsweet illusion resulted in much stronger illusion than the 180° display (t(29)= 7.6, p< .001, d = 1.39, BF_10_ = 624 000), replicating the results from Experiment 1.

The correlation averaged across all orientation-pairs for the Cornsweet illusion in the no-glare conditions was r = .73 (range .66 < r < .84 and all ps < .001, and BFs ranging from 454 to 3∙10^6^), and the corresponding average for the contrast illusion was r = .76 (range .60 < r < .90, ps < .001, and BFs ranging from 86 to 8.2∙10^8^), again demonstrating a high reliability of the PSE measures.

The correlation between the Contrast and Cornsweet illusions in no-glare conditions, averaged across all orientation pairs, was r = .32 (range .19 < r < .45 and .011< p < .31, with BFs ranging from .37 to 4.1). Although higher inter-illusion correlations were obtained than in Experiment 1 the pattern of results are similar.

To sum up, the suspicion that the anisotropy between the two horizontal orientations for the Cornsweet illusion found in Experiment 1 was due to the dim illumination from the right-hand side of the observers was confirmed. When the illumination was straight behind the observers no left-right anisotropy was obtained, but when a simulated glare was placed on the right or left side of the screen then anisotropy appeared for the Cornsweet illusion.

## Experiment 3

**Fig. 11:**
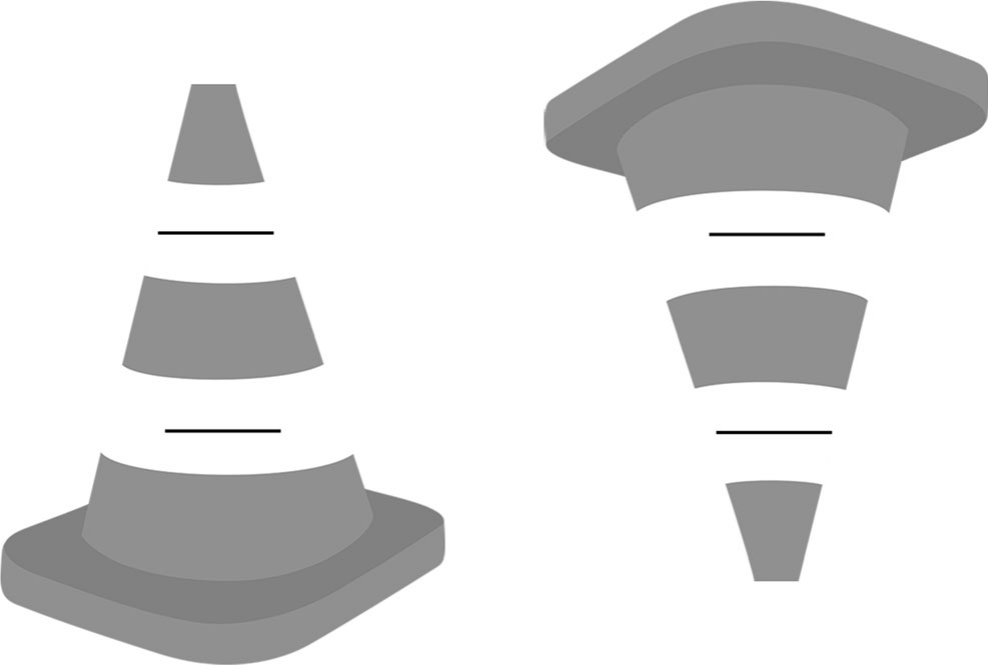
The Ponzo illusion still manifests itself although the converging lines of the silhouette of the cones (partly illusory) are not perceived to recede in depth. The horizontal line segments are perceived at the same distance from the observer. For most people, although reduced, the illusion persists when turning the display upside down

In Experiment 1 the Ponzo illusion was stronger in the upright condition than in the inverted condition. This finding supports the explanation based on our experiences of receding ground surfaces and associated depth cues such as linear perspective. Further support for the size constancy scaling theory is that the Ponzo illusion is enhanced when adding additional pictorial cues to the linear perspective. But this does not necessarily imply that the perceived depth, or depth cues, is the whole explanation of the effect. This is clearly demonstrated from variants of the Ponzo display where no illusion is seen, or even reversed Ponzo illusions are seen (Humphrey & Morgan 1965; Pressey, 1974a, b; Prinzmetal, Shimamura, & Mikolinski, 2001; Waite & Masaro, 1970). Rock (1984, p. 156) presented to the readers a line drawing illustrating a truncated pyramid (top part of the pyramid cut off) with a square base seen from its side (obliquely from above). The Rock pyramid is similar to the leftmost cone with a circular base illustrated in Fig. 11. Attached to the Rock pyramid were two vertically separated horizontal lines of equal lengths as also illustrated on the cone in Fig. 11. The converging outline contours of these objects are similar to the Ponzo inducers. Rock argued that although the converging outlines of the pyramid were not perceived as receding in depth, and the outline of the pyramid could not be utilized as cues to depth, the illusion still persists. To my knowledge, no systematic investigation of Rock’s claim has yet been made. Since in his demonstration, with the pyramid outline consisting of the converging lines not receding in depth, a preserved illusion suggests that some mechanism other than size constancy is at work here – for example, a purely stimulus-driven effect such as tilt repulsion, or assimilation and contrast, or a solely empirically driven effect from associations formed between proximal stimuli and behavioral success with no claims of veridical perception.

Figure 11 (upright cone) demonstrates, similar to the Rock demonstration, that size constancy may not be the single cause of the illusion since the illusion persists although the lines seem to be at approximately the same distance. Still, the upside-down version of this display again seems to decrease the illusion. In these displays the upper and lower horizontal lines are incompatible with a different-distance interpretation from a linear perspective. Since these effects seem modest on inspection of Fig. 11, an investigation to test these effects was performed.

### Method

In the psychology department, 32 colleagues of mine responded to a mail-based query with Fig. 11 attached and were asked if the upper or lower line looked longer or if the lines were perceived as having the same length, and if the illusion was stronger in the upright version or the upside-down version of the cone. The hypothesis that the results were due to a real difference between perceived line lengths was tested against the null hypothesis that there was no such difference. Bayes factors for binomially distributed observations were calculated using the on-line calculator at http://pcl.missouri.edu/bf-binomial.

### Results

For the upright cone, 31 observers perceived the upper line as longer, and one reported equal length (BF_10_ = 4 ∙ 10^6^ using a flat prior, p < .001). For the upside-down version, 27 out of 32 perceived the lower line as longer whereas no one reported the upper line as longer (BF_10_ = 646, p < .001). That the upright cone produced the strongest illusion was reported by 21 observers, whereas six observers reported that the upside-down version of the cone produced the strongest illusion and five reported no difference in illusion strength. Using the number of reported differences only (N=27) in the analysis, a reliable stronger illusion in upright position was found, BF_10_=16, p < .001. When using all 32 responses including those reporting no difference as the total number instead of the 27 reported differences, BF_10_ = 1, p = .025, no reliable difference between reported differences could be observed from all responses as indicated by the BF, whereas the p-values seems to support a difference. In brief, although the lines are perceived to be the same distance, the Ponzo illusion persists, but is for most observers stronger for the upright cone, again showing an up-down asymmetry.

## Discussion

Summarizing the results, upright versions (0°) of the displays resulted in stronger illusions than inverted versions (180°) as expected from up-down experience based anisotropies. Experiment 2 replicated the influence of orientation on the Cornsweet and contrast illusions, and provided evidence that the direction of illumination from the right side in the lab caused the horizontal anisotropy found for the Cornsweet illusion in Experiment 1. Experiment 3 showed that the Ponzo illusion persists although the image is not compatible with the targets being at different distances from the observer, and thus no compensation of proximal size due to distance is required. No or low correlations were obtained between magnitudes of different illusions, but within illusions the correlations were high between orientations.

The Cornsweet illusion is much stronger in the upright condition than in the upside-down condition; averages from Experiments 1 and 2 are 10.8% illusion magnitude in upright versus 2.35% illusion magnitude in the inverted condition. In the 0° condition, the Cornsweet biphasic edge is consistent with shading due to the 3D structure given the light-from-above assumption, triggering shading compensation discounting the illuminant in the process of brightness perception. In the 180° upside-down version the Cornsweet display given the light-from-above assumption is consistent with pigmentation (or a sharp ridge with the light-from-above assumption, although this is rarely reported) where no compensation for shading is required. The difference between up-right and inverted Cornsweet displays presented here is in line with the results obtained by Purves et al. (1999). In Experiment 2, the Cornsweet illusion was also influenced by manipulating the direction of simulated glare, providing additional support for the involvement of illumination direction assumptions.

The contrast illusion can also result from illumination interpretation mechanisms compensating for varying illumination. Given the light-from-above assumption, parts of objects facing upwards are more frequently illuminated directly than areas facing downwards, which are more frequently shaded and indirectly illuminated. The results from Experiments 1 and 2 support this claim, showing for the first time that the contrast illusion is stronger in the upright (0°) compared to the inverted orientation (180°). In the upright condition illusion magnitude was 28.5% and in the inverted condition it was 23.25%, measured as the average from Experiments 1 and 2. This is much less than the difference in illusion magnitude obtained by inverting the Cornsweet illusion, suggesting less influence of assumed direction of illumination on the contrast illusion. The results from Experiment 2 showed no reliable evidence for the influence of the direction of glare on the contrast illusion, which may result from the much weaker influence of assumed illumination compared to the Cornsweet illusion, as found in Experiments 1 and 2. Both the Cornsweet and the contrast illusions are considerably enhanced by strengthening the evidence for an illumination interpretation (Purves et al., 1999; Williams et al., 1998), providing additional evidence for an empirical account.

Both experience-based and stimulus-based processes have also been suggested as explanations for the Ponzo illusion. If the illusion is a case of size constancy triggered by distance cues such as linear perspective, then we expect an influence of orientation due to up-down asymmetries in our physical world. Rock (1984), however, claimed that inverted Ponzo figures have no effect on the illusion. From informal observations, he concluded that inverting the figure eliminates the impression of depth induced by the converging lines, but that the illusion remains unchanged, which suggests mechanisms other than compensation of distance when observing size. Shown here for the first time to my knowledge is that the Ponzo illusion is indeed influenced by orientation. The illusion magnitude in the upright condition was 5.6% and in the inverted condition it was 3.2%. The cause is likely orientation anisotropies in perceived slant since we are more familiar with ground planes receding in depth than ceiling surfaces, and have rich experiences with linear perspectives from roads and railway tracks on the ground, than corresponding ceiling stimuli. If the number of cues to distance is increased in a Ponzo display, the illusion seems to increase in magnitude, supporting the size-distance compensation account (Leibowitz et al., 1969). Support for an anisotropy of experienced slant direction comes from natural image analysis showing that the probability distribution of environmental slants in different directions (backward and forward slant) is highly anisotropic (Inagami & Kaneko, 2011), and studies in the lab have come to the same conclusion about perceived slant (Poom et al., 2007). An additional point regarding the Ponzo illusion deserves attention: The size of objects in the upper part of visual space are perceived as slightly larger than objects in the lower part (Luckiesh, 1922, p. 44; Robinson, 1972, p. 104, as mentioned in Higashiyama & Yamazaki, 2016). The reason might be that objects closer to the inferred horizon appear farther away than objects more distant from the horizon. The linear perspective and the proximity to horizon cue come into conflict when turning the classical Ponzo illusion upside down, and may dampen the influence of orientation on perceived size. This cannot, however, explain the Ponzo illusion in the reversed condition of Experiment 3, where the lower line seems longer than the upper one despite no required distance-size compensation from a linear perspective. The more subjective response format used in Experiment 3 (due to lack of resources) compared to the adjustment method used in Experiments 1 and 2 may be a caveat for conclusions drawn from comparisons between experiments.

A Bayesian framework can successfully account for some of the contextual influences on lightness perception (Allred & Brainard, 2013) and the Cornsweet illusion (Brown & Friston, 2012). These models use intuitively reasonable priors of likely surface reflectances and illuminations. For example, that illumination varies more slowly over space than surface reflectance. Similar assumptions were made in a vector model of color and brightness perception (Bergström, 1977). These assumptions reflect veridical structure and illuminations in our environment, and may be learnt based on hard-wired mechanisms, or a combination of these. The wholly empirical theory, on the other hand, supposes that our perceptions represent an ordering of visual stimuli according to accumulated past experiences of proximal stimuli without the involvement of priors of distal configurations such as shadings (Purves et al., 2015). In this view, perception, as exemplified by perceived brightness, relies on learnt frequencies of occurrences of target patches of different lightness in dark and bright backgrounds, respectively, without the need to rely on prior assumptions about shading in our environment. Luminance values in neighboring areas in an image are highly correlated in natural images so dark targets in dark backgrounds and light targets in light backgrounds have statistically occurred more frequently than vice versa (Yang & Purves, 2004). Ordering all possible targets’ luminance against the dark and the luminous backgrounds, respectively, provides a measure of each target’s rank against each background, where the 0th percentile and the 100th percentile correspond to minimum and maximum perceived brightness, respectively. The same target luminance can have different ranks, and thus are experienced as different, in different context. The ranking theory shares similarities with anchoring, where the most luminous part in a specific framework takes the value of “white,” although proponents of the anchoring theory do not refer to frequencies of past proximal stimuli as an origin of the anchoring process. A wholly empirical account of the spatial upright versus upside-down asymmetry of the Ponzo illusion could be based on statistical occurrences of converging lines and associations with depth in real images, where the inverted Ponzo illusion is less associated with depth than non-inverted displays, and this occurs irrespective of whether depth is seen or not. Therefore, the outline of the cone suffices to trigger depth-size compensation mechanisms, although the target lines are seen as being presented at the same distance.

We should not ignore the evidence against the empirical explanation, from previous published results, as the sole cause of illusions. Some of the results presented here actually add to these results. Todorović (2006) presented evidence against illumination compensation mechanisms as an explanation for brightness illusions by showing that shadow-incompatible images may result in both Cornsweet and contrast illusions. Also, it should be noted that the original demonstration of the Cornsweet illusion as illustrated in Fig. 1C (topmost) is actually also shadow incompatible, a shadow-compatible interpretation would suggest that the illumination comes from all directions or involves multiple light sources spread around the stimulus. Instead of an illumination interpretation mechanism, Todorović suggested that local gradients seem to be involved to produce these illusions, engaging mechanisms of lateral inhibition with small and large field response profiles, as suggested by Hong and Grossberg (2004). In addition, the results from Experiment 3, where Ponzo illusions were obtained even when no difference in distance is apparent from the inducers, i.e. the stimulus is incompatible with an interpretation of a distance-difference, similar to Todorović (2006), demonstrates that brightness illusions occur despite stimuli being shadow incompatible. The involvement of mechanisms involved in producing the Ponzo illusion other than just a compensation for distance, such as tilt-contrast as proposed by Prinzmetal et al. (2001), could explain this result and explain why a reversed illusion appears when increasing the angle between inducing lines, as found by Pressey (1974a). The involvement of multiple processes in producing the illusion may explain that although the Ponzo illusion is rapidly established, a more time-consuming processing is required before the full integration of context information is accomplished (Schmidt & Haberkamp, 2015), and that no correlation between illusion magnitudes between illusions was obtained here, or by others (Grzeczkowski et al., 2017). Thus, when considering all evidence including the influence of orientation, explanations of these illusions seem to require the involvement of multiple mechanisms.

Within illusions, individual magnitudes of illusions were highly correlated between orientations. A pattern emerged for the Cornsweet and Ponzo illusions where the strongest correlations were obtained between nearby orientations rather than for orientations far apart; no such influence of angular separation was obtained with the Cornsweet illusion (Experiment 1). No or weak correlations were observed between the magnitudes of different illusions, which is in line with Grzeczkowski et al.’s (2017) report where they couldn’t find any correlations between a number of various illusions tested. Even susceptibility to simultaneous contrast across dimensions, with a few exceptions, is not correlated across dimensions, although the phenomenon is similar (Bosten & Mollon, 2010). One possible reason for the lack of any common factor across illusions may be that empirical influences may vary across different types of stimuli, and across individuals, due to genetic and/or environmental variability, and perceptual learning from past experiences may be very specific and do not transfer across similar stimuli (Grzeczkowski et al., 2017). Another reason, as mentioned above, might be that each illusion relies on involvement of multiple different mechanisms. Support for the involvement of different mechanisms is reported dissociations, where prolonged fixation enhances the Cornsweet illusion (Todorović, 1987), whereas brief presentations enhance the classical simultaneous contrast (Kaneko & Murakami, 2012) and various other simultaneous contrast effects (Kaneko et al., 2017). In addition, Kaneko et al. (2018) presented evidence for separate fast and slow processes mediating simultaneous contrast for brief and long flashes.

A recently developed deep-learning algorithm that incorporates hard-wired center-surround connections with classical and extra-classical receptive fields accounts for both contrast and assimilation in contextual induction phenomena (Mély, Linsley, & Serre, 2018). Such hard-wired and experience-based models of perception do not exclude one another, and both may contribute to perceptual experience. In this view, phylogenetic processes drive development in a functional direction by slowly evolving hard-wired mechanisms such as lateral inhibition, and more complex interactions. Ontogenetic processes, such as learning from experiences, contribute at later stages, and all processes push in a common direction to increase functionality of perceiving animals. The results presented here show that orientation of the displays influences illusions, and provide evidence for the involvement of assumptions about the world, or statistical occurrences of proximal stimuli. Hard-wired stimulus-driven processes such as lateral inhibitions and assimilation must be added to experience-based explanations in order to fully account for existing demonstrations of brightness and geometrical illusions.
